# Efficient biodiesel production from oleic and palmitic acid using a novel molybdenum metal–organic framework as efficient and reusable catalyst

**DOI:** 10.1038/s41598-022-14341-4

**Published:** 2022-06-20

**Authors:** Arash Ghorbani-Choghamarani, Zahra Taherinia, Yunes Abbasi Tyula

**Affiliations:** 1 Department of Organic Chemistry, Faculty of Chemistry, Bu-Ali Sina University, Hamedan, 6517838683 Iran; 2 Department of Chemistry, Faculty of Science, Ilam University, Ilam, Iran

**Keywords:** Catalyst synthesis, Sustainability

## Abstract

In this study, metal–organic framework based on molybdenum and piperidine-4-carboxylic acid, was synthesized through a simple solvothermal method and employed as an effective catalyst for biodiesel production from oleic acid and palmitic acid via esterification reaction. The prepared catalyst was characterized by XRD, FTIR, TGA, DSC, BET, SEM, TEM, ICP-OES, X-ray mapping and EDX analysis. The resulting Mo–MOF catalyst exhibit a rod-like morphology, specific surface area of 56 m^2^/g, and thermal stability up to 300 °C. The solid catalyst exhibited high activities for esterification of oleic acid and palmitic acid. Moreover, the catalyst could be simply recovered and efficiently reutilized for several times without significant loss in its activity, also obtained results revealed that metal–organic framework could be used for the appropriate and rapid biodiesel production.

## Introduction

Due to increasing environmental pollution, global warming caused by fossil fuels, many studies are seeking for develop renewable energy technologies. In this context, biodiesel are considered to have the most potential to reduce the amount of particulate matter^1^, CO_2_^2^, and greenhouse gas emissions^3^ due to high octane number^4^, and low viscosity^5^ that makes it a promising and economically feasible alternative to common fossil fuels^6^. Biodiesel is a renewable energy that can be produced by transesterification of triglyceride in biomass-oil (such as vegetable oil and animal fat) with methanol^7^. Traditional biodiesel production methods utilize homogeneous conditions in the presence of bases or an acid catalyst such as sulfuric acid and sodium hydroxide^8^.These homogeneous systems suffer from limitations such as corrosion of reactors, difficult recovery and recyclability of catalyst and environmental pollution^9,10^. In this context, heterogeneous catalysts provide more advantages over homogeneous catalysts, such as recyclable^10,11^, ease separation-purification process^12^, high glycerol purity^13^, and not corrosive^14^. To date, numerous solid acid and base catalysts with tunable structural and surface functionalities have been offered, and many of them shows high catalytic activity for biodiesel production yield. Heterogeneous solid base catalysts usually provide higher rates compared with the acid counterparts under milder reaction conditions. However, they cannot be directly employed for oil having more than 2 wt% FFA due to side reactions, such as saponification and hydrolysis, and decrease both the catalyst activity and the ester yield^15^. Hence, solid acid catalysts are applied when dealing with low-quality or non-edible vegetable oils containing significant quantities of FFAs and water^15^. Molybdenum compounds have been recognized as versatile catalysts, because of the ability of this metal to be on the solid surface in different oxidation states, ranging from Mo^6+^ to metallic Mo (Mo^0^)^16^.

Anhydrous sodium molybdate^17^, bulk MoO_3_^18^, silica MoO_3_/B-ZSM-5^19^ molybdenum supported on alumina^20^, silica, silica-alumina, and titania^21,22^, as well as carbon^23^ have been used as esterification and transesterification catalysts for biodiesel production from several oils, including waste oil. Gandía et al., described the application of bulk and Al_2_O_3_-supported molybdenum oxide for the production of biodiesel from Oil. Control experiments showed that bulk MoO_3_ is very active for both transesterification and esterification reactions, but it suffered from severe molybdenum leaching in the reaction medium.

Compared to bulk MoO_3_, alumina-supported MoO_3_ leads to a more efficient utilization of the active phase and enhanced stability towards molybdenum leaching by the reaction medium^24^. In this study, we have been introduced a novel MOF as a highly efficient separable catalyst for biodiesel production from oleic acid and palmitic acid via an esterification reaction. In recent years, metal–organic frameworks (MOFs) are attracting increasing attention due to their important properties such as controllable composition^25^, large surface area^26,27^, thermal stability^28^, flexibility, and easy preparation^29^. MOFs are constructed from SBU connected by organic linkers to form extended coordination networks. The linkers widely used in MOFs are rigid organic chelators such as amino acid, terephthalic acid, and polycarboxylate ligands. Many factors affect the activity of MOFs, such as the organic ligand, solvent type, particle size, and metal sort. Metal–organic frameworks (MOFs) known as coordination polymers, undergoing study by many researchers for advanced applications, including such as catalysis^30–32^, separation^33,34^, gas storage^35^, carbon dioxide capture^36^. MOFs mainly have their adjustable nano-structures and porous properties. However, as a good carrier, MOF also has its inherent catalytic performance. Moreover, the MOF-based functional catalytic materials show great potential in biodiesel production and other relevant biorefineries. In Table 1, typical heterogeneous bifunctional catalysts were chosen to compare with MOF-based bifunctional catalysts for biodiesel production. These studies revealed that the MOF with amino groups (Brønsted base) showed high catalytic activity and mild conditions for biodiesel production than another heterogeneous acid–base catalyst (Table 1).

**Table 1:** Comparison between bifunctional catalysts based on MOF and other proposal materials for biodiesel production.

Entry	Catalyst	Reaction condition	Oil	Yield (%)	Ref.
1	UiO-66(Zr)-NH_2_	39: 1, 4 h, 60 °C, 6 wt%	Oleic acid	97.3	^37^
2	NH_2_-MIL-101(Cr) Sal-Zr	10: 1, 4 h, 60 °C, 4 wt%	Oleic acid	74.1	^38^
3	AILs/HPW/UiO-66-2COOH	35: 1, 6 h, 110 °C, 10 wt%	Soybean	95.27	^39^
4	Fe_3_O_4_@HKUST-1	30: 1, 3 h, 110 °C, 1.5 wt%	Soybean	92.3	^40^
5	Polymeric acidic ILs-based Fe_3_O_4_	30: 1, 5 h, 130 °C, 8 wt.%	Oleic acid	96.2	^41^
6	Polymeric sulfonated ionic liquid based Fe_3_O_4_/SiO_2_	35: 1, 6 h, 120 °C, 9 wt%	Soybean	93.6	^42^
7	CaO/MZSM-5(CH-U)	12: 1, 3 h, 110 °C, 4 wt.%	Rapeseed	90.4	^43^
8	CaO-La_2_O_3_	25: 1, 3 h, 160 °C, 3 wt%	Soybean/ Jatropha Jatropha	98.7	^44^
9	A-15/PVA	29: 1, 8 h, 65 °C, 25 wt%	Waste cooking	98	^45^
10	Zn8@Fe-C_400_	40: 1, 4 h, 160 °C, 7 wt%	Jatropha	100	^46^

**Figure 1:**
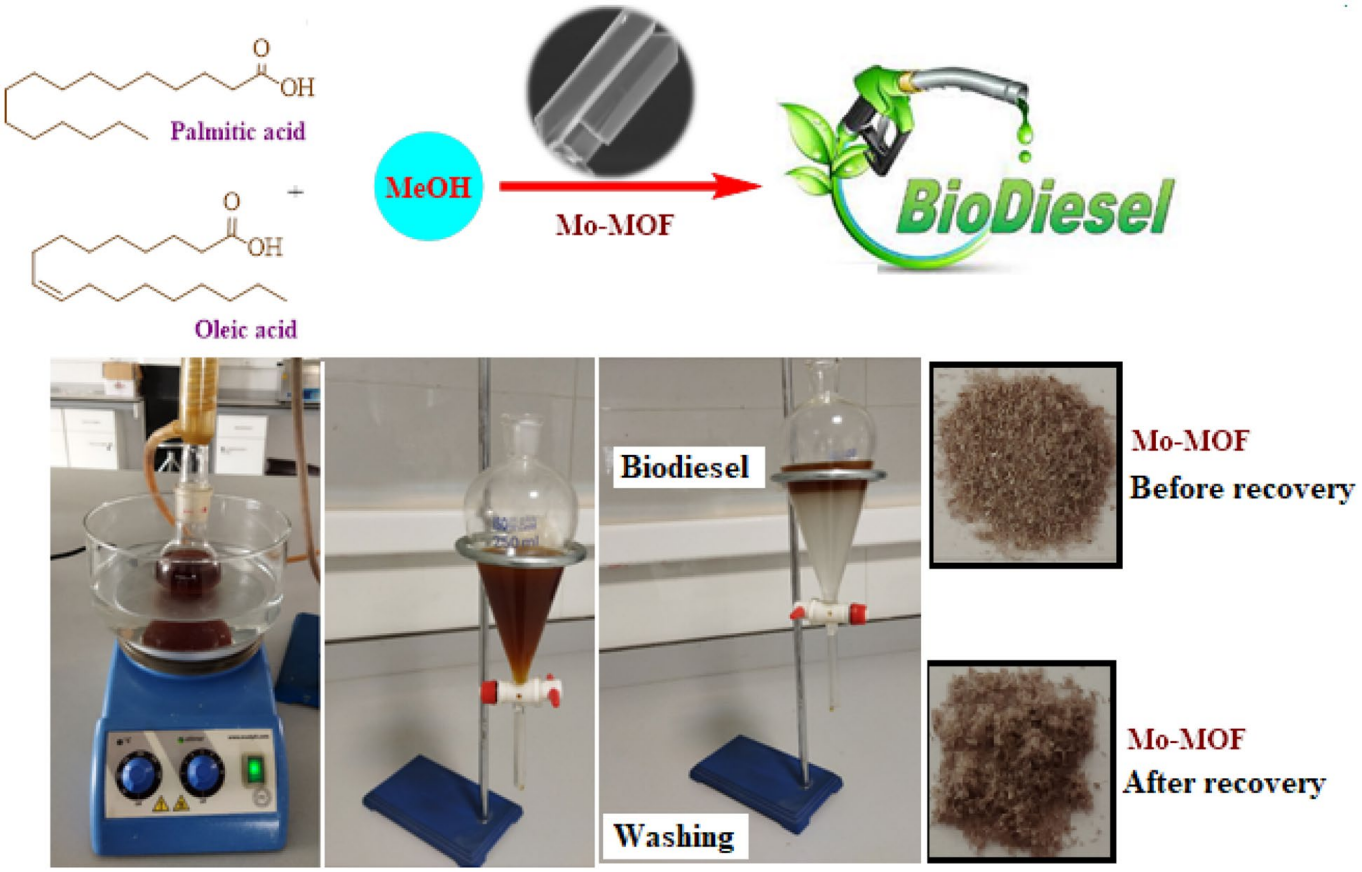
The application of metal–organic framework for biodiesel production.

To the best of our knowledge, there have been few papers referred to preparation of Mo–MOFs^47–52^. In this study, the catalytic performance of metal–organic framework based on molybdenum and piperidine-4-carboxylic acid was employed in biodiesel production through the esterification of oleic acid and palmitic acid with methanol (Fig. 1).

**Scheme 1:**
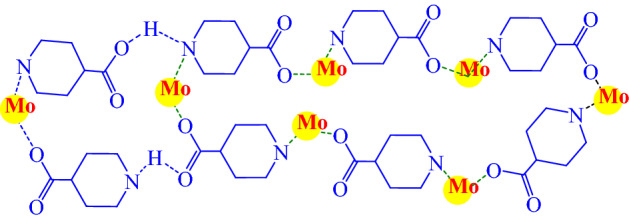
The topological structure of Mo–MOF.

The structure of a network is affected by several factors such as building blocks, solvent, temperature, pH, and so on. Also, its topology is mainly dependent on the connectivity and the symmetry of the metal ions (or metal clusters) and organic nodes. We find the rod-MOF topology [Rod MOFs are metal–organic frameworks in which the metal-containing secondary building units consist of infinite rods of linked metal-centered polyhedral], according to the present approach, the metal ions are connected to the framework by the organic SBUs (Scheme 1).

## Experimental section

### Solvothermal synthesis of Mo–MOF

Mo–MOF was prepared by mixing Na_2_MoO_4_ with 4-piperidinecarboxylic acid (2:1 molar ratio M:L) in 20 mL of the DMF/H_2_O (18:2; v/v) and then magnetically stirred at 200 rpm for a 30 min at room temperature. Subsequently, the mixture was transferred to a Teflon-lined stainless steel autoclave and heated isothermally at 170 °C for 24 h. After, the autoclave was gradually cooled to room temperature. Finally, the resultant brown crystal was filtered by vacuum filtration followed by washing with ethyl acetate and dried at 60 °C in vacuum oven for 6 h (Scheme 1).

### Biodiesel production

The catalytic activity of Mo–MOF was assessed for esterification reactions of oleic acid and palmitic acid by mixing oil (1 mol), methanol (13 mol), and MOF (300 mg) mixed in a round-bottom flask. The mixture was heated at 60 °C for 4 h. After the reaction is done, the catalyst was separated by centrifuges (5000 rmp), and the excess methanol was removed from the upper liquid phase using rotary evaporation completely. The extracted organic phase was further washed with distilled water to remove the residual impurities by decantation and dried with anhydrous Na_2_SO_4_.

### Characterization of Mo-based metal–organic frameworks

**Figure 2:**
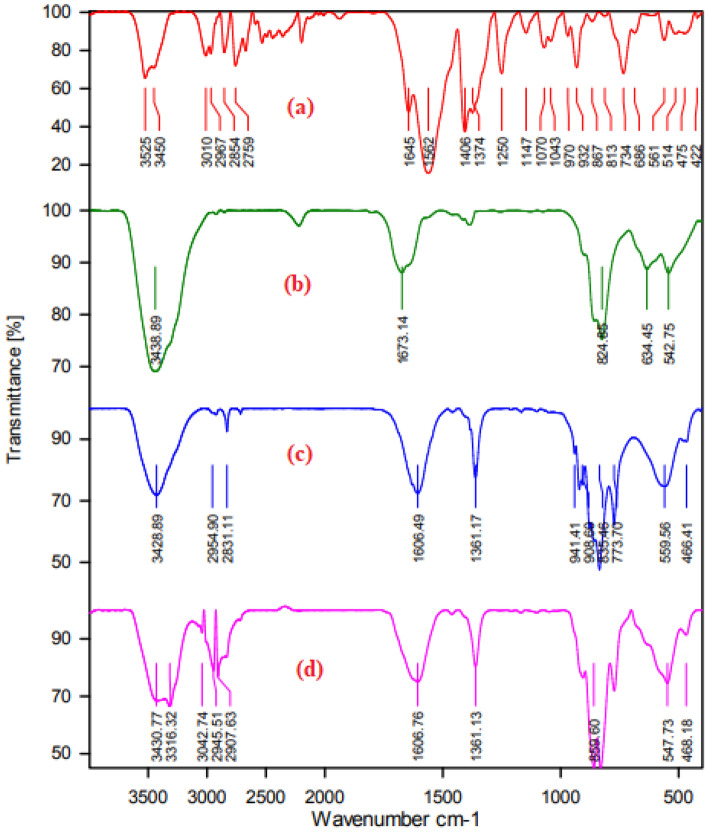
FT-IR spectra of 4-piperidinecarboxylic acid (a) Na_2_MoO_4_·2H_2_O (b) Mo-based metal organic framework, before recovery (c) Mo-based metal organic framework, after recovery (d).

The catalytic system has been studied by FTIR, XRD, BET, SEM, TEM, EDX, ICP-OES, TGA, DSC, and EDS elemental mapping. Moreover, Fourier transforms infrared (FT-IR) spectroscopy has been introduced for the quantitative analysis of Mo-based metal–organic frameworks. Figure 2 provides the FT-IR spectra of Na_2_MoO_4_. 2H_2_O (a), 4*-*piperidinecarboxylic acid (b), Mo-based metal–organic framework (c). FT-IR spectrum of 4-piperidine carboxylic acid exhibited two peaks at 3525 and 3450 cm^−1^ corresponding to stretching vibrations of –OH and –NH groups, respectively. The peak at 1645 cm^−1^ was attributed to the carboxylic (-COO) group of piperidine. The peaks at 2854 and 2967 cm^−1^ are corresponded to –C–H stretching of the methylene group. The strong peak of C–N stretching was observed at 1406 cm^-1^. Moreover, –OH bending and –N–H wagging of secondary amine were observed at 970 and 686 cm^−1^, respectively (Fig. 2a). The stretching vibration of Fig. 2b, was detected as a strong Mo–O stretch in the [MoO_4_]^2−^ tetrahedrons at 824–634 cm^−1^ and additional weak peaks of the Mo–O bending mode around 500 cm^−1^. Moreover, the band at 3439 cm^−1^ is the characteristic of the stretching modes of the O–H bond of Na_2_MoO_4_. 2H_2_O. It can be observed that the carboxylic (−COO) group of piperidine carboxylic acid in the Mo-based metal–organic framework at 1606 cm^−1^ disappeared, indicating that the attachment of the carboxylic (−COO) group to the metal (Fig. 2c). It is worth noting that the C–N stretch band, which was present at 1406 cm^−1^ in the spectrum of 4-piperidine carboxylic acid, which is absent in the spectrum of the Mo-based metal–organic framework (Fig. 2c).

**Figure 3:**
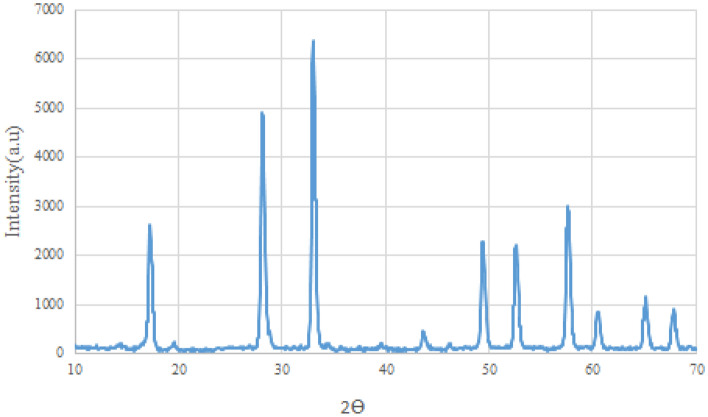
XRD pattern of Mo-based metal–organic framework (MOF).

Powder X-ray diffraction was used to determine the chemical composition and crystal structure of the typical synthesized products. Obviously, Mo–MOF exhibits a series of sharp peaks, indicating its good crystallization. The characteristic peaks are at 28.17, 33.12, 49.37, 52.7, 57.2, 65.17, and 68.2, which are identical to representative reference^47,48^, (Fig. 3)*.* All of these characteristics indicate that Mo–MOF was successfully synthesized.

**Figure 4:**
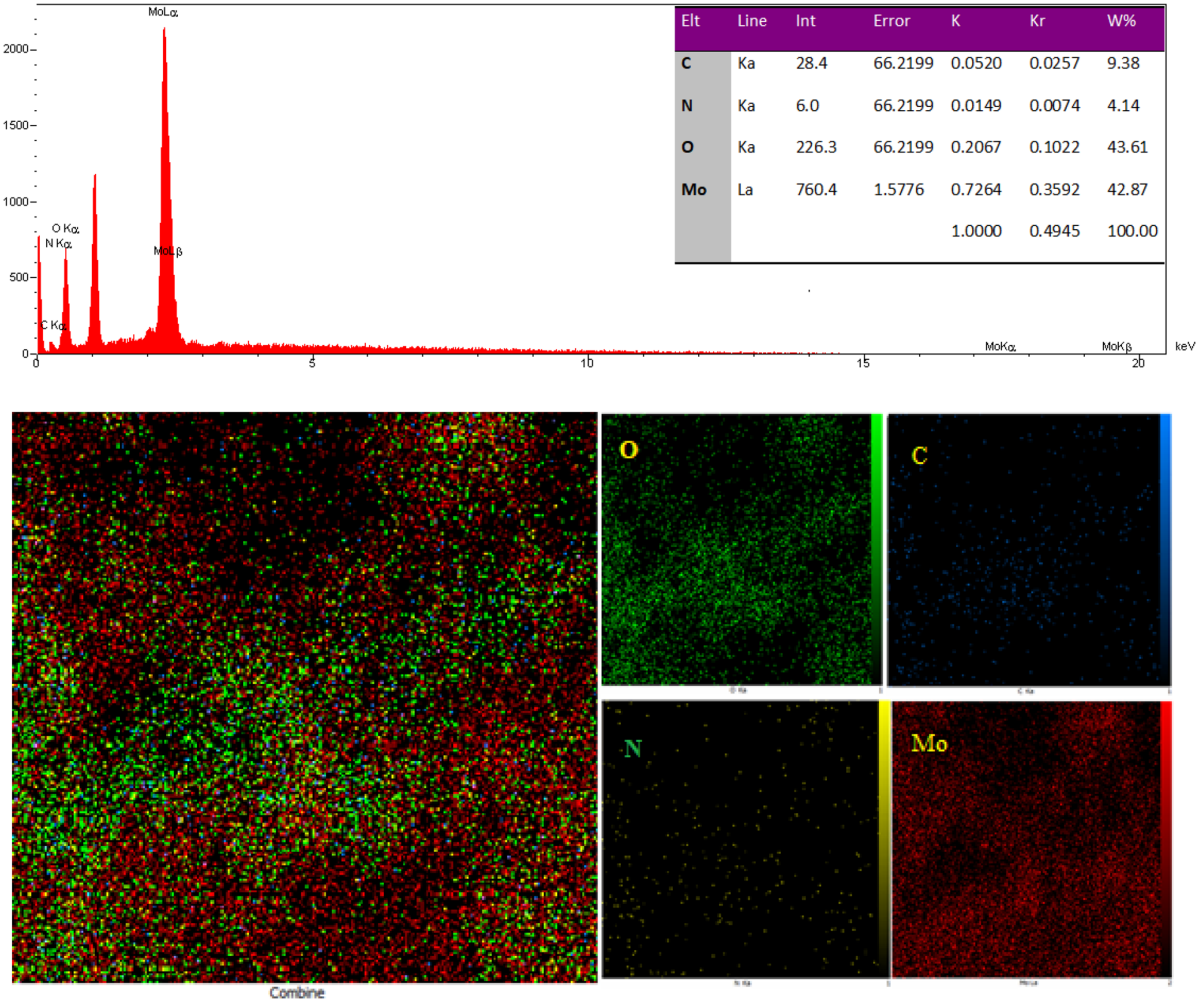
X-ray mapping analysis and EDX spectra for Mo–MOF.

Figure 4 shows the X-ray mapping and EDX analysis of Mo–MOF. The EDX spectrum indicates the percentage of index elements in Mo–MOF (C = 9.38%, N = 4.14%, O = 43.61% and Mo = 42.87%). Mapping analysis, reveals the occurrence of Mo as metallic and C, N, O as non-metallic constituents are homogeneously distributed within the metal–organic framework (MOF).

**Figure 5:**
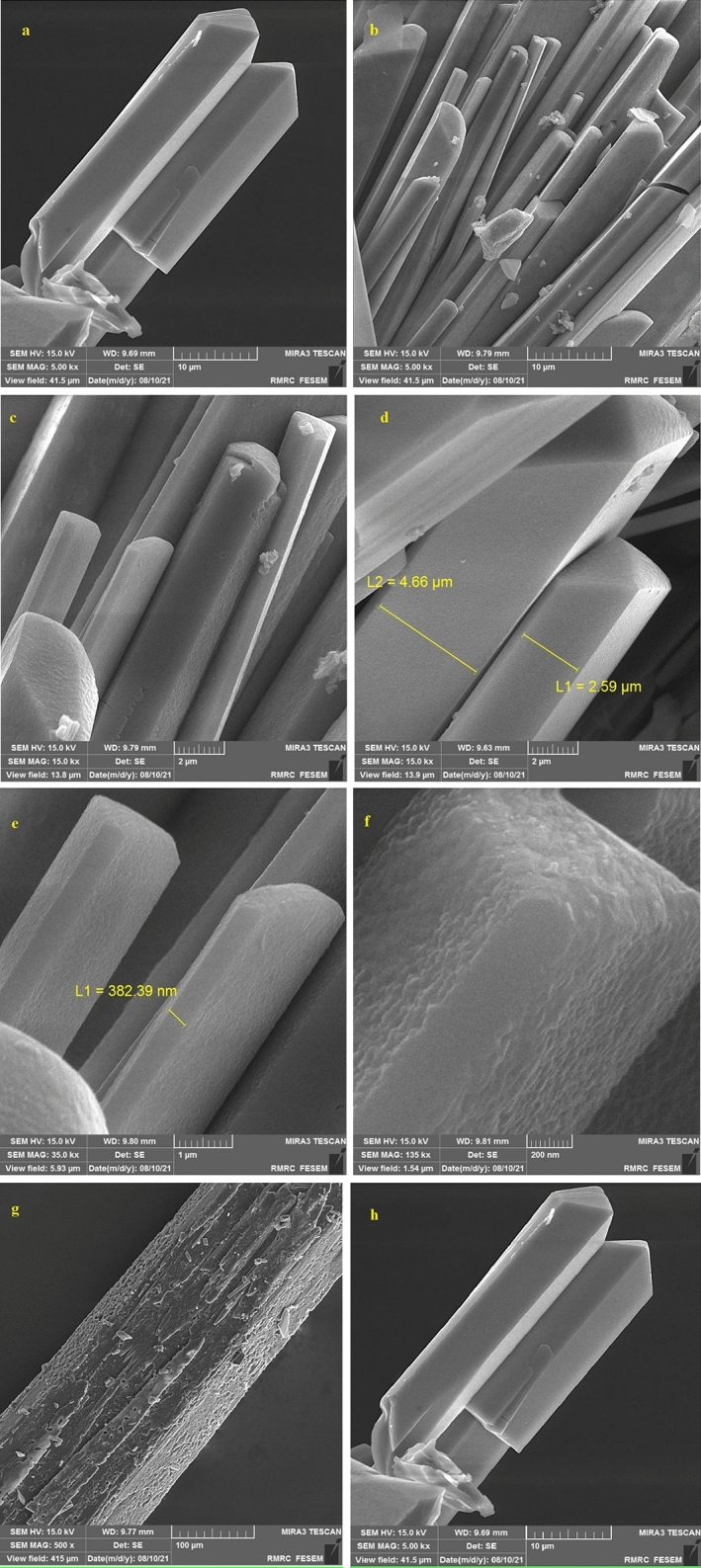
SEM images of Mo-metal–organic framework (MOF).

The morphologies of Mo–MOF are monitored by scanning electron microscopy. The SEM images (Fig. 5) revealed that morphology of the product featured a rod-like structure with a well-defined plane facing along the transversal direction at approximately 500 nm–2 µm. The inset in Fig. 5a displays the edge of a bar with the typical cleavage for monoclinic structures (inset Fig. 5a).

**Figure 6:**
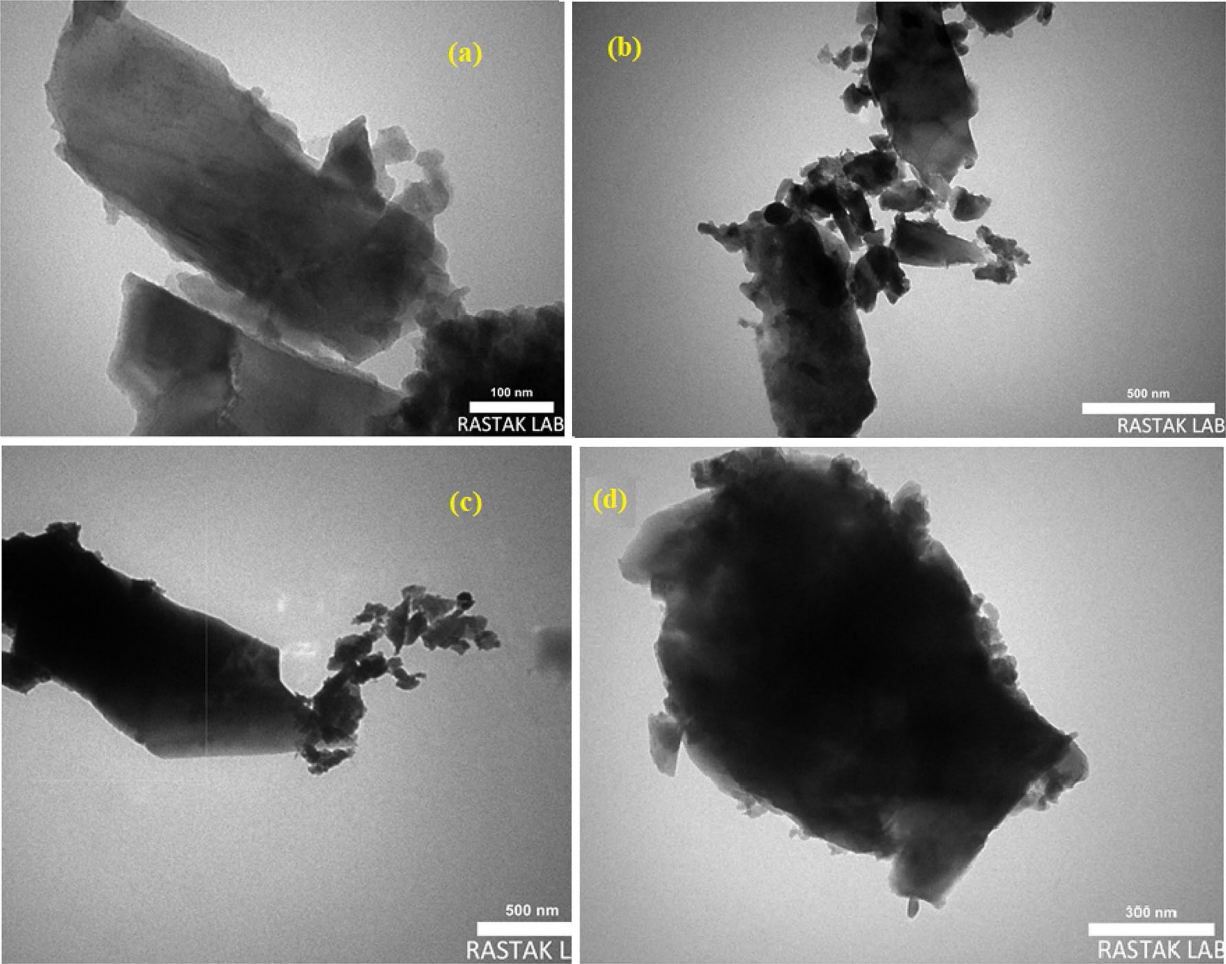
TEM images of Mo-metal–organic framework (MOF).

The structure and morphology of the Mo-metal–organic framework (MOF) have been studied by TEM. Figure 6 shows that the particles have a nanorod-like structure.

**Figure 7:**
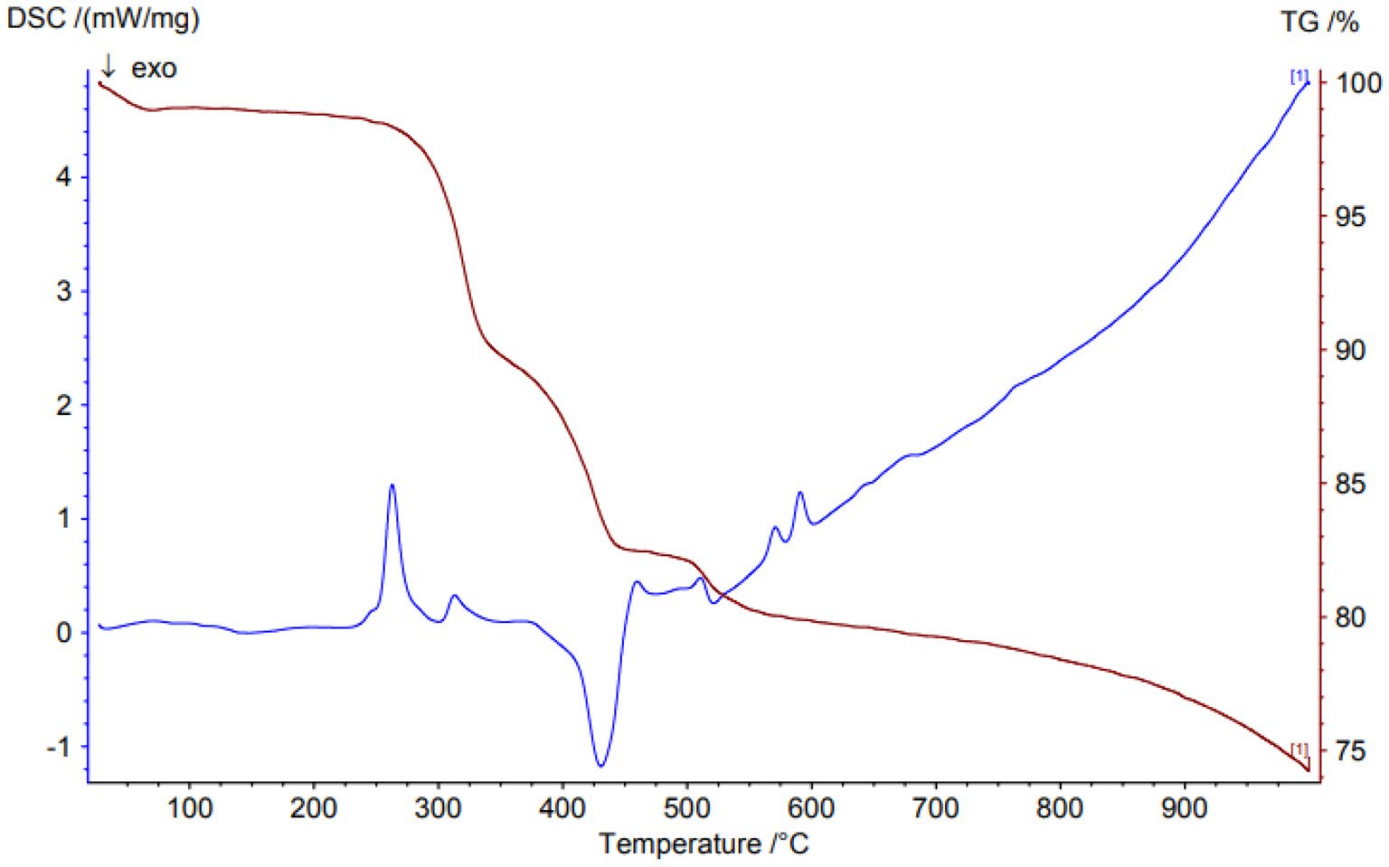
TGA of Mo-metal–organic framework (MOF).

TGA (Thermo-gravimetric analysis) was performed to investigate the thermal stability of the synthesized materials and was carried out from room temperature to 1000 K by using a TGA analyzer at a heating rate of 10 K/min under air atmosphere (Fig. 7). The result demonstrated thermodynamic stability of the materials up to 300 °C. The weight loss was observed in two steps: the first weight loss (3%) occurred in the range of 100 °C related to the loss of physically adsorbed H_2_O molecules and organic solvents. The second weight loss (19%) between 330 and 600 °C is corresponding to the decomposition of the organic species. The DSC shows two endotherms at 270 and 330 °C which corresponds to the successive release of water and DMF molecules from the host channels. The DSC analysis also displays an exothermic after complete loss of functional groups of guest molecules and coordination modes at approximately 420 °C which show that the compound undergoes phase transformation.

**Figure 8:**
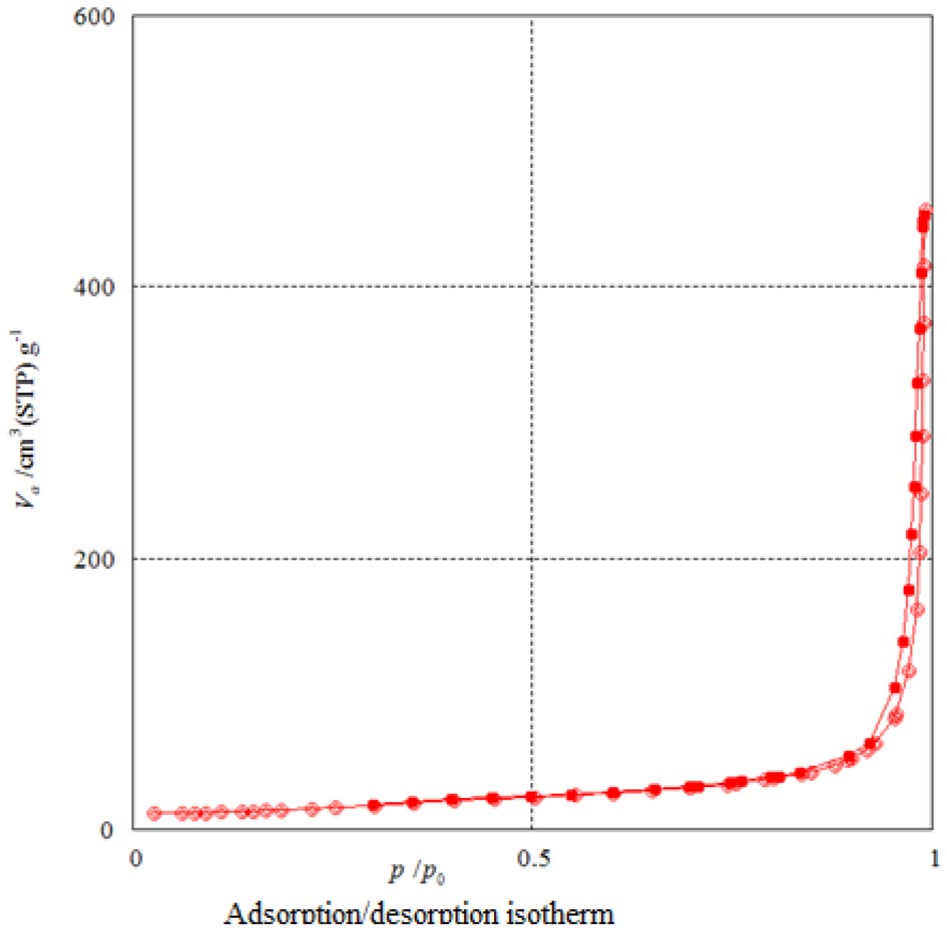
Nitrogen adsorption–desorption isotherms of Mo–MOF.

**Figure 9:**
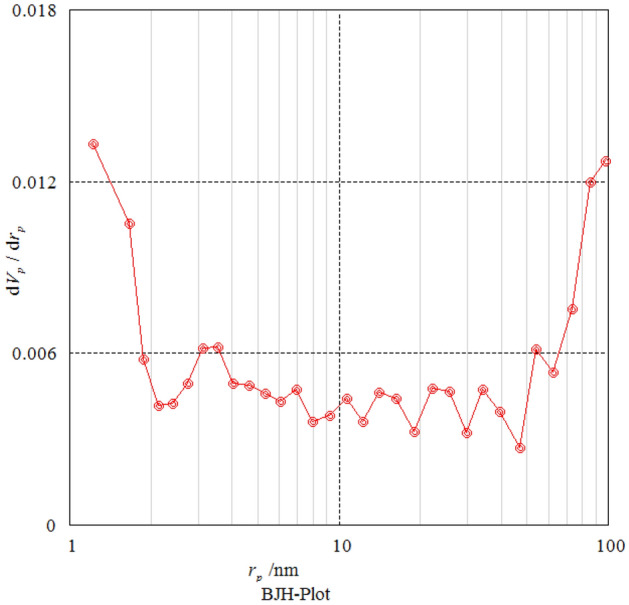
BJH pore size distribution for Mo–MOF.

The texture and porosity of the Mo–MOF was quantified by measuring the nitrogen adsorption isotherm (Fig. 8). The results show that according to the IUPAC classification of adsorption isotherms, the N_2_ isotherm resembles the type III having sharp adsorption capacity while indicating the presence of broader pore size distributions, and narrower mesopores, and wider micropores. According to The Brunauer–Emmett–Teller (BET) method, the specific surface area and pore volume were estimated to be 56 m^2^g^−1^ and 12. 98 cm^3^g^−1^. The BJH pore size calculations using the adsorption branch of the nitrogen isotherm indicate a micropore peak at about 1.66 nm of diameter for Mo–MOF (Fig. 9)*.*

### Material performance

In this study, the catalytic properties of the investigated Mo–MOF systems were performed by the esterification of oleic acid and palmitic acid with methanol. The biodiesel production was optimized using Mo–MOF as catalyst (100–300 mg), and various oleic acid/alcohol molar ratios at different temperatures. According to the obtained results, the yield of the reaction decreased with the catalyst amount decreasing. This may be due to the decrease in the total sorption surface area available to adsorbates resulting from the overlapping of active sites. According to these results, the maximum biodiesel production was achieved by300 mg of catalyst. A maximum conversion of 95% (oil to ester) was achieved for the temperature of 60 °C. The molar ratio between methanol and oil was considered to be 13:1 in this study for the completion of the esterification process (Table 2).

**Table 2:** Experimental data for the optimized yield of biodiesel from oleic acid(A) and palmitic acid(B) with methanol in presence of Mo–MOF.

Entry	Catalyst amount (mg)	Temperature (°C)	Methanol to oil molar ratio (mol/mol)	Biodiesel yield (%)^a^	
				A	B
1	300	25	10:1	73	65
2	300	40	10:1	78	71
3	300	60	10:1	88	87
4	200	60	10:1	81	70
5	100	60	10:1	41	35
6	300	60	13:1	95	90
7	300	60	15:1	95	90
8	60	60	7:1	38	31
9	300	60	5:1	23	21
10	300	60	3:1	15	9

^a^Reaction time(4 h), Isolated yield.

### Fuel properties of biodiesel

The physicochemical properties of the biodiesel, such as density, viscosity, flash point, pour points, and ash was determined according to the standard ASTM methods. The resulting biodiesel was pure and showed excellent physical and chemical properties meeting international standards. Kinematic viscosity is both cold flow and critical property of an oil to be used in compression engines and it represents the degree of resistance to flow offered by the fluid. The viscosity of biodiesel should be in the range of 1.9–6.0 mm^2^/s. The Kinematic viscosity of biodiesels made from palmitic acid and oleic acid are calculated to be 3.9 and 4.06 °C, respectively, which indicates these parameters have met the appropriate standard of ASTM D445. The flashpoint is the lowest temperature at which a liquid can form an ignitable mixture in air near the surface of the liquid. From Table 3, the flashpoint of biodiesels made from palmitic acid and oleic acid are measured to be 140, and 160 °C, respectively which is in agreement with ASTM D92. The pour point represents the lowest temperature at which a liquid will begin to flow. The pour point of biodiesels made from palmitic acid and oleic acid is calculated to be 12 and − 3 °C, respectively, which agrees with ASTM D97. The cloud point is the temperature at which wax crystals begin to form in a liquid as it is cooled. The cloud point of the biodiesel from palmitic acid and oleic acid were 17 and − 1 °C, respectively. Cloud points of biodiesels were almost within the ASTM D2500 standard range. Ash content: when organic compounds are decomposed at high temperatures (500–600 °C), the leftover residue is called ash. The approved limits of the carbon and ash residue after biofuel ignition are 0.01 wt% according to ASTM D-482, respectively. The obtained ash residues of the biodiesel from palmitic acid and oleic acid were between 0.004 and 0.002 wt% using Mo–MOF. These values are comparatively lower than those obtained in the case of petroleum fuel.

**Table 3:** Fuel properties of biodiesel.

Entry	Characteristics	Result palmitic acid	Result oleic acid	Unit	Test method
1	Kinematic viscosity at 40 °C	3.9	4.06	cSt	ASTM D445
2	Flash point	140.2	160	°C	ASTM D92
3	Pour point	12	− 3	°C	ASTM D97
4	Cloud point	17	− 1	°C	ASTM D2500
5	Ash content	0.004	0.002	wt%	ASTM D482

### Comparison

In order to show efficiency of the catalytic activity of Mo–MOF, we compared our results for the esterification of reaction of oleic acid has been shown in Table 4. It is evident that our protocol shows excellent catalytic activity in terms of yield of product.

**Table 4:** Comparison of results for Mo–MOF in esterification of reaction of oleic acid.

Entry	Catalyst	Conditions	Time (h)	Yield (%)^a^	Refs.
1	HClSO_3_–ZrO_2_	The molar ratio of methanol to oleic acid being 8, 100 °C	12	100	^53^
2	F^−^-SO_4_^2−^/MWCNTs	The molar ratio of methanol to oleic acid being 12:1 at 65 °C	6	90	^54^
3	[BHSO_3_MIM]HSO_4_	The molar ratio of methanol to oleic acid being 4:1 at 130 °C	2	97.7	^55^
4	ZrFe-SA-SO3H	The molar ratio of methanol to oleic acid being 12:1 at 90 °C	4	92.7	^56^
5	Picolinic acid modified 12-tungstophosphoric acid	The molar ratio of methanol to oleic acid being 10:1, 100 °C	5	100	^57^
6	Mo–MOF	The molar ratio of methanol to oleic acid being 13:1, 65 °C	4	95	This work

^a^Isolated yields.

### Catalyst reusability and catalytic performance comparison of Mo–MOF

**Figure 10:**
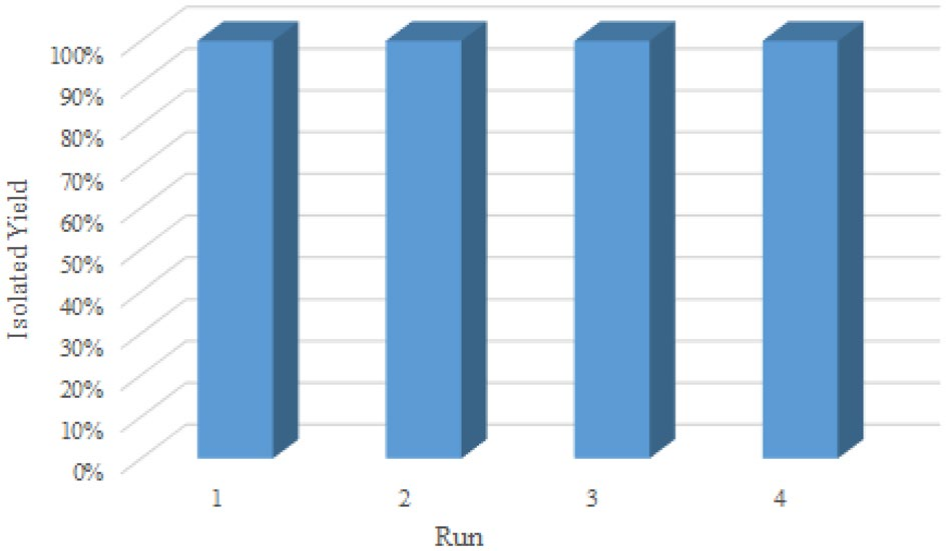
Recyclability of Mo–MOF in the esterification of oleic acid with methanol.

Reusability emerged as a very important factor to determine the robustness of a heterogeneous solid catalyst and its commercial potential, particularly from economic and practical viewpoints. Filtration and centrifugation are two of the methods traditionally employed on a laboratory scale to allow handling, separation, recovery, and recycling of heterogeneous catalysts. Furthermore, the MOFs are often suitable as highly recyclable catalytic systems with easy and efficient recovery of catalysts through simple filtration or centrifugation. In this regard, the catalytic stability of Mo–MOF was investigated by the esterification of oleic acid. After the reaction was completed, the catalyst was separated using centrifugation. Then, it was washed with methanol and dried entirely at 80 °C. The dried catalyst was reused in the second reaction under optimum reaction conditions (Fig. 10). The catalyst was recycled efficiently for four cycles and the catalyst showed almost constant activity in the esterification of oleic acid (92% after the 4th recycle was obtained). The retrieved catalyst after four recycles maintained its original framework structure, as evidenced by FT-IR technique. The FT-IR spectrum of the recovered Mo–MOF indicates that this catalyst can be recycled without any change in its structure (Fig. 2d). Moreover, the nature of the catalyst was also determined by investigating the leaching of the molybdenum species by using ICP-AES analysis. ICP-OES analysis exhibited very low leaching of molybdenum species upon reuse. Based on the results from the ICP-OES analysis, the amount of molybdenum in the fresh catalyst and the recovered catalyst after four runs was 0.077 mol g^−1^ and 0.072 mol g^−1^ respectively, which this observation was attributed to the strong interactions of heteroatoms in the piperidine-4-carboxylic acid with molybdenum. Moreover, the results confirmed the heterogeneous nature of the catalysis.

## Conclusion

In summary, a novel Mo–MOF was prepared by solvothermal method. Structural features of the material were ascertained by several analytical techniques like XRD, FTIR, TGA, DSC, BET, SEM, TEM, ICP-OES, X-ray mapping, and EDX analysis. Interestingly, obtained solid catalyst exhibits more highly catalytic activity in the esterification of oleic acid and palmitic acid with methanol. The influential reaction parameters, including the methanol/oil molar ratio, temperature, and catalyst amount, are optimized. The physicochemical properties of the biodiesel, such as density, viscosity, flash point, pour points, and ash was determined according to the standard ASTM methods. Control experiments showed that the fuel properties of prepared biodiesel are found to meet the international biodiesel standards. Moreover, the catalyst could be simply recovered and efficiently reutilized several times without significant loss in its activity, also obtained results revealed that this metal–organic framework could be used for the appropriate and rapid biodiesel production. This strategy can produce a solid catalyst with catalytic site accessibility, which has a good application future in the field of green chemistry.

## Supplementary Information

Supplementary Information.
