# Supplementary material for: Efficient biodiesel production from oleic and palmitic acid using a novel molybdenum metal–organic framework as efficient and reusable catalyst
Source: Sci Rep. 2022 Jun 20;12:10338. doi: 10.1038/s41598-022-14341-4 (PMC9209509; doi:10.1038/s41598-022-14341-4)

**Preparation, Characterization and Catalytic Application of Metal-Organic Framework Based on Molybdenum and Piperidine-4-Carboxylic Acid as a Novel Catalyst for Biodiesel Production from Oleic acid and Palmitic acid**

Arash Ghorbani-Choghamarani,^a*^ Zahra Taherinia,^b^ [Yunes Abbasityula](https://www.sciencedirect.com/science/article/abs/pii/S0040402013009289#!)^b^

Address correspondence to ^a^ Department of Organic Chemistry, Faculty of Chemistry, Bu-Ali Sina University, Hamedan 6517838683, Tel: +988138282807, Fax: +988138380709, Iran; E-mail: [arashghch58@yahoo.com](mailto:arashghch58@yahoo.com) or a.ghorbani@basu.ac.ir; ^b^ Department of Chemistry, Faculty of Science, Ilam University, Ilam, Iran.

**NMR data**

**Methyl palmitate**:^1^H NMR (CDCl_3_, 400 MHz) δ= 0.089 (t, *J*=0.088, 3H), 1.25(s, 24H), 1.62(quintet, *J*= 1.60, 2H), 2.32(t, *J*= 2.30, 2H), 3.66(s, 3H).FT-IR (KBr) mmax/cm^-1^ : 586, 726, 781, 884, 989, 1175, 1377, 1465, 1744, 2853, 2922.

**Methyl oleate**:^1^H NMR (CDCl_3_, 400 MHz) δ= 0.091 (t, *J*=0.089, 3H), 1.28(s, 12H), 1.31(s, 8H), 1.63(quintet, *J*= 1.60, 2H), 2.03(t, *J*= 2.01, 2H), 2.37-2.33(m, 4H), 3.68(s, 3H), 5.39(q, *J*=5.37, 2H).FT-IR (KBr) mmax/cm^-1^ : 436, 591, 723, 873, 976, 1016, 1173, 1366, 1455, 1743, 2857, 2926.

.


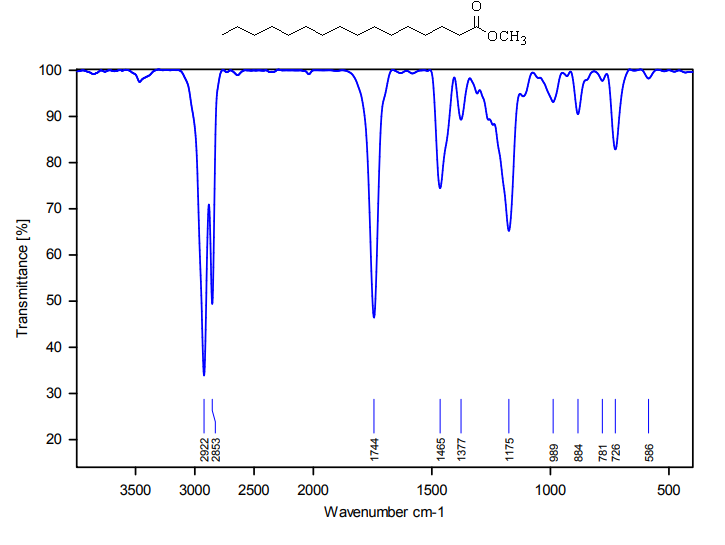


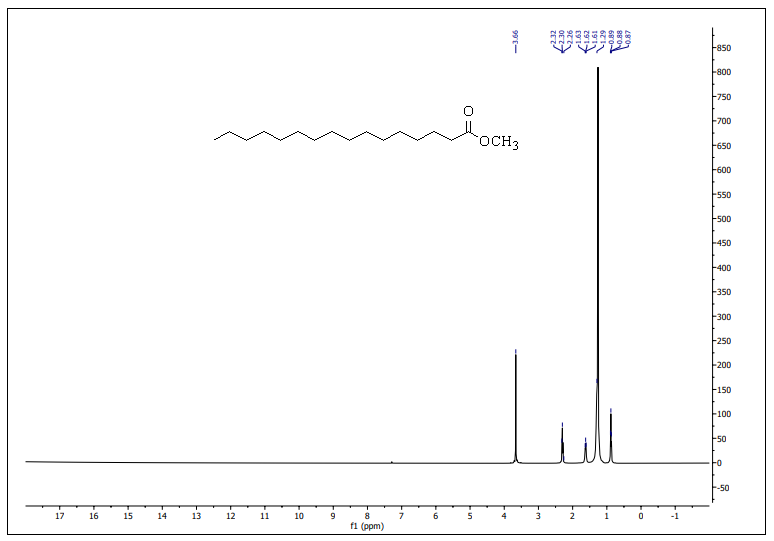


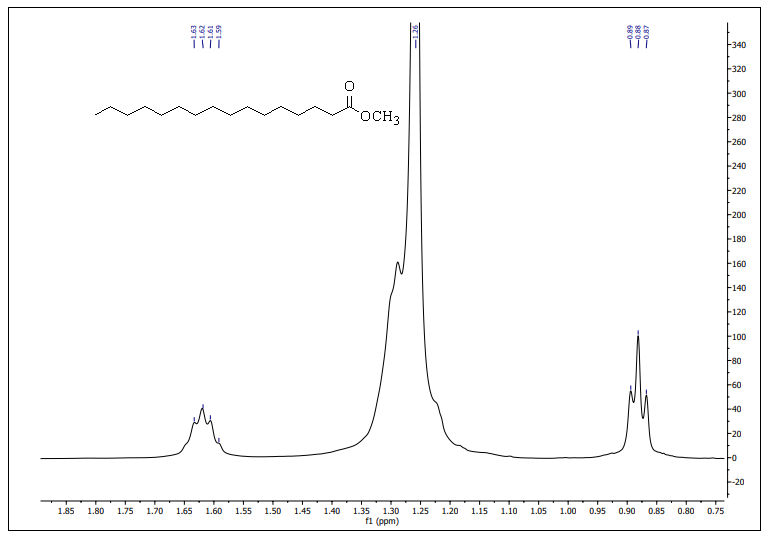


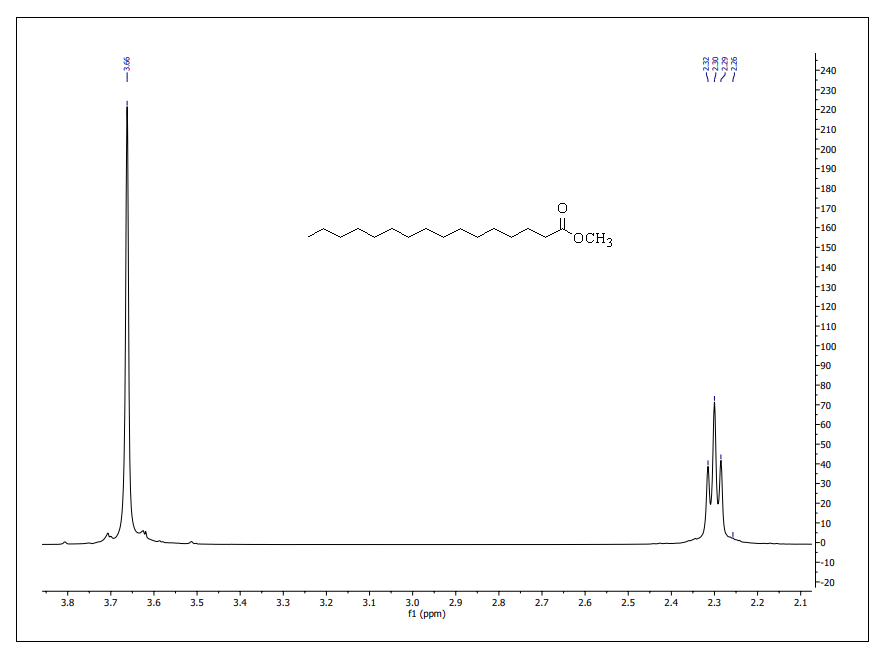


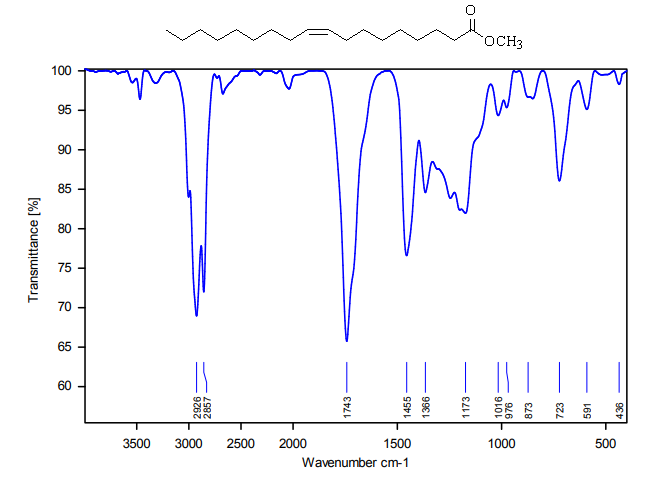


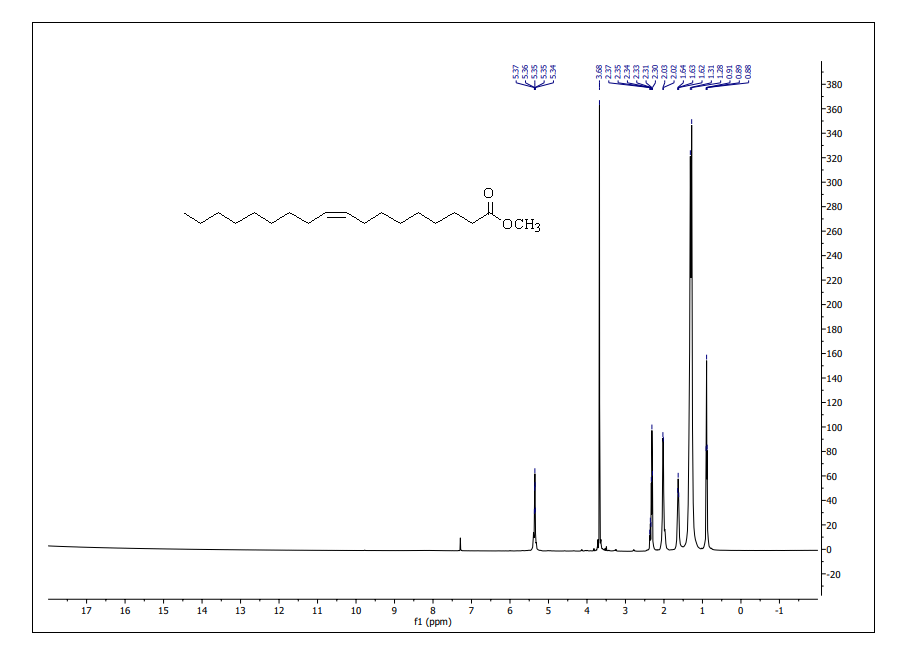


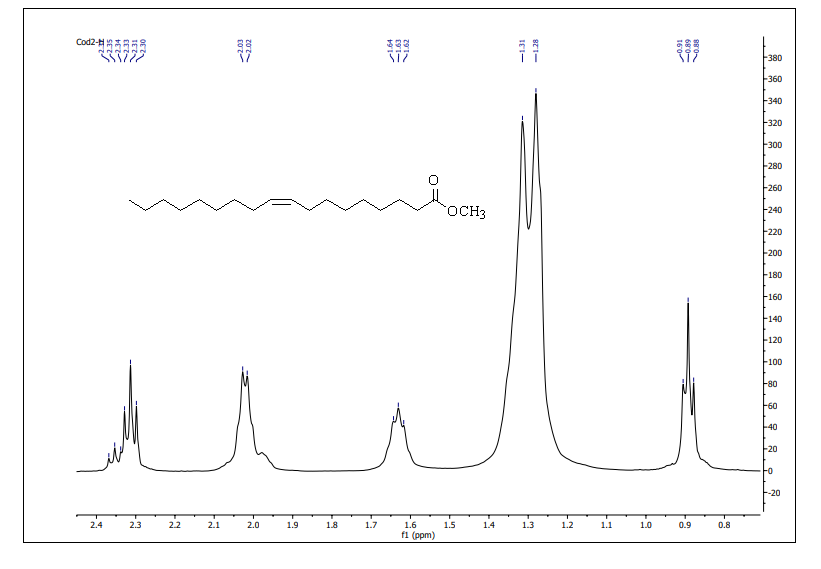


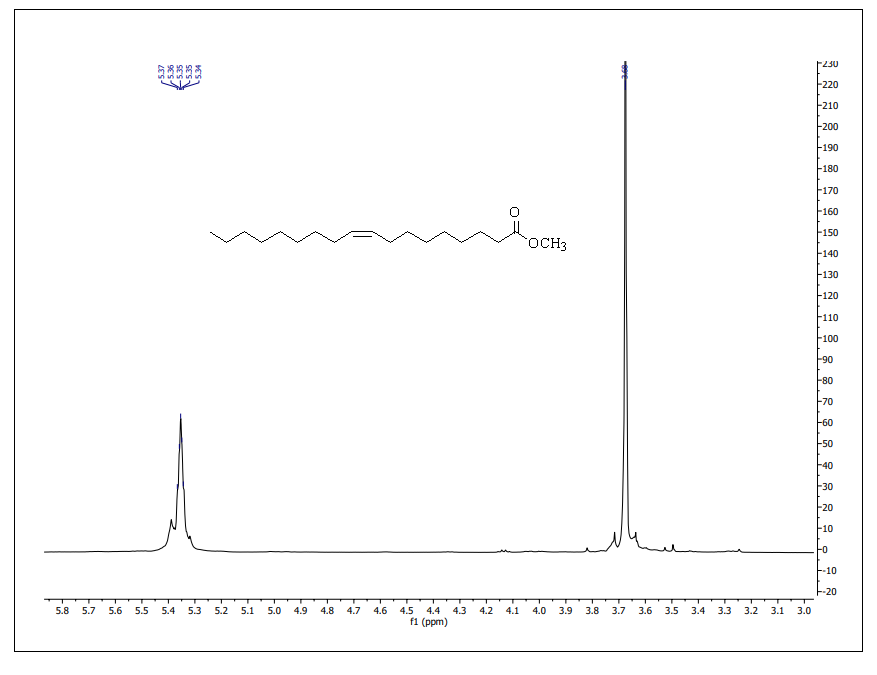

Supplement: Supplementary file 1 — Supplementary Information. [file 41598_2022_14341_MOESM1_ESM.docx]
